# 肺癌术后声音嘶哑的病因、评估与综合管理

**DOI:** 10.3779/j.issn.1009-3419.2026.106.21

**Published:** 2026-07-20

**Authors:** Pengfei MEN, Jialin LIANG, Feng ZHANG, Xiaolong WANG

**Affiliations:** ^1^475000 开封，河南大学第二临床学院（门鹏飞，梁甲林）; ^1^The Second Clinical College of Henan University, Kaifeng 475000, China; ^2^河南大学淮河医院胸外科（张锋，王晓龙）; ^2^Department of Thoracic Surgery, Huaihe Hospital of Henan University, Kaifeng 475000, China

**Keywords:** 肺肿瘤, 声音嘶哑, 喉返神经损伤, 声带麻痹, 纵隔淋巴结清扫, 综合管理, Lung neoplasms, Hoarseness, Recurrent laryngeal nerve injury, Vocal fold paralysis, Mediastinal lymph node dissection, Comprehensive management

## Abstract

肺癌术后声音嘶哑是胸外科常见但易被低估的围手术期功能性并发症。其不仅影响发声质量，还可能累及吞咽与气道保护功能，从而增加误吸、肺部感染及康复延迟风险。医源性喉返神经损伤是主要病因，且与纵隔淋巴结清扫密切关联；其中左下气管旁淋巴结区域为典型高危解剖区。喉返神经损伤既可由直接机械性损害引起，也可因牵拉与热损伤等间接因素导致神经功能障碍。与此同时，气管插管相关喉部机械性损伤亦可表现为术后声音嘶哑，需与神经源性病因鉴别。本文总结病因谱与危险因素，并概述术后评估要点与预防策略，包括精细解剖、减少热损伤及合理使用术中神经监测。未来研究应推动前瞻性与标准化证据，以明确不同干预的最佳时机与适用人群，并完善胸外科综合管理路径，提升患者术后生活质量。

## Abstract (en)

Hoarseness after lung cancer surgery is a common but often underestimated perioperative functional complication. Beyond impairing voice quality, it may involve swallowing dysfunction and compromised airway protection, thereby increasing the risk of aspiration, pulmonary infection, and delayed recovery. Iatrogenic recurrent laryngeal nerve injury is the major cause, closely associated with mediastinal lymph node dissection, with the left lower paratracheal lymph node station as typical high-risk anatomical areas. Nerve injury results from both direct mechanical trauma and indirect mechanisms such as traction and thermal spread. In addition, intubation-related laryngeal trauma can also present as postoperative hoarseness and should be differentiated from neurogenic causes. This review summarizes the etiology and risk factors, outlines postoperative evaluation and preventive strategies, and discusses the role of meticulous dissection, minimized thermal injury, and appropriate intraoperative neuromonitoring. Future prospective studies are needed to standardize outcomes and clarify the optimal timing and target populations for interventions, ultimately improving comprehensive perioperative management and patients’ quality of life.

随着胸腔镜与机器人辅助手术的普及，肺癌外科治疗在提升根治效果的同时，对围术期功能保护的要求日益提高。术后声音嘶哑是具有代表性的功能性并发症之一，在纵隔淋巴结清扫后尤应引起重视。对于左侧肺癌手术患者，喉返神经在主动脉弓下及气管食管沟区域走行，易在淋巴结清扫与组织游离过程中受到牵拉、压迫或热损伤；而右侧上纵隔操作亦可能因牵拉及局部血供干扰导致神经功能障碍^[1-3]^。Nakamura等^[3]^报道2例右上叶肺癌患者在接受右上叶切除联合右侧上纵隔淋巴结清扫后出现左侧喉返神经麻痹，提示右侧纵隔操作亦可能通过纵隔牵拉、移位及间接机械应力影响喉返神经功能。既往研究^[2,4-6]^显示，肺癌术后声带麻痹并非少见，且与术后咳嗽无力、吞咽障碍及误吸相关，可能增加肺部感染风险并延长住院时间；其发生率在不同研究^[6,7]^中报道为6.0%-31.0%；其中左侧手术相关风险相对更高，最高可达31%^[6]^。来自人群队列的证据^[8]^亦提示，单侧声带麻痹与肺炎风险升高有关；与此同时，气管插管相关喉部损伤同样可导致术后声音嘶哑，使临床病因判断更具挑战^[9]^。

在实际诊疗中术后声音嘶哑常被视为“可自愈的不适”而延迟评估与干预，导致部分患者错过嗓音康复或早期声门闭合改善的窗口期；而对存在明显误吸风险者，延误处理可能带来更严重的呼吸系统并发症。因此，有必要基于胸外科情境建立更清晰的病因识别思路、评估路径与分层管理策略。本文聚焦医源性喉返神经损伤这一关键病因，系统梳理肺癌术后声音嘶哑的病因谱与危险因素，概述术后评估要点，并总结预防和阶梯式治疗策略，以期为胸外科围术期管理与多学科协作提供参考。

## 1 病因：以医源性喉返神经损伤为核心

### 1.1 医源性喉返神经损伤是主要病因

肺癌术后声音嘶哑最重要的病因是医源性喉返神经损伤，其机制通常为多因素叠加。纵隔淋巴结清扫过程中，尤其在主动脉弓下、气管食管沟的左下气管旁淋巴结（left lower paratracheal lymph node station, 4L）区域，神经可能遭受牵拉、压迫、误夹或切割等直接机械性损伤^[2,3]^。除直接损伤外，能量器械在神经邻近区域使用所致热扩散亦可影响神经传导功能；相较之下，减少高热源暴露或采用更温和的分离与止血策略更有利于神经保护^[10,11]^。此外，局部小血管网破坏、过度骨骼化分离以及持续牵张，可能诱发节段性缺血并造成迟发性神经功能障碍；术后水肿、渗血或局部压迫亦可导致一过性症状加重。

临床上喉返神经损伤除表现为声音嘶哑外，常伴发声无力、气息声、有效咳嗽能力下降、饮水呛咳及吞咽不适，部分患者还可出现术后肺部并发症风险增加及住院时间延长^[4,6]^。

### 1.2 需重视与气管插管相关损伤的鉴别

并非所有术后声音嘶哑均由喉返神经损伤所致。气管插管相关喉部损伤同样常见，机制包括气囊压迫、插管摩擦、杓状软骨脱位以及黏膜水肿或血肿等^[9]^。其他专科术后声音嘶哑的经验亦提示，术后声音嘶哑病因具有异质性，不能简单等同于神经损伤^[12]^。此类患者多在拔管后即刻出现声音异常，常伴喉痛、异物感或显著发声费力；而喉返神经损伤相关症状可在术后数小时至数日逐渐显现，且更容易合并呛咳与无效咳嗽。

因此，对术后持续声音嘶哑患者，不宜仅凭手术方式或经验推断病因，而应尽早行喉镜检查，以明确声带运动状态、黏膜情况及杓状软骨位置，从而完成与插管相关机械性损伤的鉴别，并为后续分层干预提供依据^[9,13]^。

## 2 解剖学基础与围术期危险因素

### 2.1 左侧喉返神经是术中保护重点

左侧喉返神经绕行主动脉弓后返向上行，于气管食管沟内走行，路径较长、位置较深，与4L关系密切，因此在左侧肺癌手术中更易受到损伤^[1-3]^。当需暴露主动脉弓下区域并实施4L清扫时，神经常处于持续牵拉、压迫及热损伤的高风险环境。与右侧手术相比，左侧相关操作在神经识别、牵拉控制及工作空间暴露方面更具挑战性。

除常规解剖关系外，左侧喉返神经的分支方式及其与周围结构的毗邻关系亦存在个体差异，解剖变异本身即是增加术中损伤风险的重要因素^[14]^。此外，如右位主动脉弓、异常锁骨下动脉及非返性喉返神经等少见变异，均可能进一步提高术中误伤概率^[1,15]^。因此，术前影像学评估除用于指导肿瘤切除及淋巴结清扫方案外，还应用于评估纵隔大血管及神经结构的可能异常走行与解剖风险，从而提升高危患者的术前风险分层与围术期准备。

### 2.2 手术范围与器械使用方式直接影响损伤风险

系统性纵隔淋巴结清扫涉及更多神经邻近操作，尤其在上纵隔和4L区域，可能增加术后声音嘶哑或喉返神经功能障碍风险^[7]^。若过度强调清扫彻底性而忽视神经边界保护，可能导致暂时性甚至持续性神经功能障碍。需要强调的是，4L清扫并非单纯“高风险操作”，其在左侧非小细胞肺癌中的分期价值和临床意义已得到多项研究支持^[16-18]^。因此，临床决策的重点并非简单回避4L区域，而是在保证肿瘤学完整性的前提下优化神经保护策略。

器械选择及其使用方式同样是可干预的重要因素。已有回顾性分析及综述^[10,11]^表明，在喉返神经邻近区域进行淋巴结清扫时，减少高热源暴露、谨慎使用能量器械，有助于降低声带麻痹发生风险。因此，在高危区域宜尽量采用直视下锐性分离、分步止血及短时低强度能量输出策略，避免长时间、近距离热传导造成神经损伤。除此之外，再手术、局部粘连明显、肿瘤与纵隔组织关系致密以及术者经验不足等因素，也可能进一步增加术中神经损伤风险。

## 3 术后评估与分层策略

### 3.1 早期识别与高危人群筛查

根据分层评估结果，后续处理路径按附图1（http://www.lungca.org/files/2026s111/2026s111-s1.pdf）所示在观察随访、嗓音治疗、注射喉成形及进一步外科干预之间进行阶梯式选择。肺癌术后声音嘶哑的临床意义不应仅限于发声质量下降。此外，术后声音嘶哑病因具有异质性，不能简单将处理流程概括为所有持续性声音嘶哑均立即行喉镜检查；应结合手术侧别、纵隔淋巴结清扫范围、术中操作情况、症状严重程度及吞咽、气道保护功能进行风险分层与处置决策^[6,7]^。对于部分患者，声音改变可能伴随声门闭合不全、咳嗽与吞咽保护功能受损，从而增加误吸及肺部感染风险；队列研究提示单侧声带麻痹与肺炎风险升高有关^[5,8,13]^。因此，一旦出现持续性声音改变或伴饮水呛咳及排痰无力等表现，应尽早进入规范评估流程，避免以“短暂刺激”解释后延误分层处置。

高危患者尤其需要提高警惕，包括左侧肺癌伴4L清扫者、术中神经暴露或牵拉明显者、局部粘连严重者，以及术后伴有明显呛咳或吸入性肺炎倾向者。对上述人群进行早期识别，有助于及时完成病因判断与风险分层，并为后续干预创造条件^[2,4,6,13]^。

### 3.2 喉镜检查是诊断核心

对高危患者可考虑低阈值开展术后喉镜评估；至于是否常规筛查，现有证据仍有限^[13,19]^。对于存在高危因素的患者，如左侧肺癌手术、4L清扫、术中明显牵拉或热能量器械使用、淋巴结融合粘连、新辅助治疗后纤维化、术后饮水呛咳或误吸风险高、排痰无力、肺部感染倾向或氧合下降等，应进一步降低喉镜检查阈值^[2,6,7,14,20]^。若上述患者术后声音嘶哑持续超过2周，或即使未超过2周但合并吞咽障碍、呛咳或误吸或呼吸道症状，应尽早行喉镜评估以明确声带运动情况、判断是否存在声带麻痹或声门闭合不全^[6,7,21]^。相反，对于低危患者（未行高风险纵隔淋巴结清扫、术中未见明显神经牵拉或热损伤风险、声音嘶哑程度轻且无饮水呛咳、误吸表现，并呈逐渐改善趋势者），可在密切随访基础上观察至术后4-6周；若症状持续、加重或出现吞咽及呼吸相关表现，则应进一步行喉镜检查^[21,22]^。此外，需强调急危重症情形，尽管双侧喉返神经同时损伤罕见，但一旦发生可能导致严重声门闭合不全及气道梗阻。若术后出现呼吸困难、喘鸣或严重误吸并伴低氧，应立即进行喉镜评估以明确声带运动与气道状态，并及时启动相应急救与多学科处理路径，避免等待常规分层随访导致病情进展。喉镜能够直接观察声带活动度、声带位置、声门闭合情况及喉部黏膜状态，是确认单侧或双侧声带运动障碍的核心检查手段^[13,19]^。

对肺癌术后患者而言，喉镜检查的意义不仅在于判断是否存在声带麻痹，更在于完成病因鉴别。通过喉镜可识别声带固定、黏膜水肿、肉芽形成及杓状软骨位置异常等改变，从而区分喉返神经损伤、插管相关喉部损伤及杓状软骨脱位等不同病因，并为后续治疗方案提供依据^[9,13]^。

### 3.3 嗓音量化评估与患者报告结局

术后评估不应局限于解剖或运动学异常的识别，还应结合嗓音功能受损程度进行综合判断。临床上可联合应用嗓音声学分析、空气动力学指标及患者报告结局量表，对发声障碍进行量化评估^[13,23]^；其中，嗓音障碍指数（voice handicap index, VHI）等量表能够反映嗓音问题对交流能力、社会参与及生活质量的影响，而客观声学参数则有助于动态观察病程演变及治疗反应^[13,23]^。这一评估框架有助于实现从“声带活动异常”到“功能损害程度”的转化判断，从而提高治疗决策的针对性。

### 3.4 喉肌电图、吞咽功能及误吸风险评估

当需鉴别神经源性麻痹与环杓关节固定、杓状软骨脱位，或评估神经功能恢复潜力时，喉肌电图具有重要补充价值^[13]^。对于术后较早期即提示恢复前景较差的患者，喉肌电图有助于尽早明确病变性质，并优化后续干预时机。与此同时，吞咽功能及误吸风险评估亦应纳入围术期管理。对伴有饮水呛咳、痰液潴留、反复低热或肺部感染的患者，应高度警惕隐匿性误吸，并尽早联合耳鼻喉科、康复医学科及言语治疗团队开展评估与干预^[4,13]^。必要时可联合吞咽筛查、吞咽造影或纤维鼻咽喉吞咽功能检查，以进一步评估气道保护功能并指导饮食与康复方案^[13]^。因此，本节所述评估主要用于在文章3.1部分所阐述的分层框架基础上进一步明确吞咽与气道保护风险及神经恢复潜力，从而细化后续观察与阶梯式干预策略（附图1， http://www.lungca.org/files/2026s111/2026s111-s1.pdf）。

### 3.5 动态随访与干预时机判断

声音嘶哑的自然转归存在显著个体差异：部分患者可能在数周至数月内逐步恢复，而恢复趋势与持续时间决定是否进入更高阶干预窗口^[7,13,24]^。因此，评估应基于动态随访而非一次性判断。

随访内容应包括症状变化、喉镜下声带运动恢复情况、吞咽及误吸表现、肺部感染发生情况，以及患者生活质量变化。对于改善趋势明确者，可继续观察并配合保守治疗；而对症状持续、误吸风险高或职业用声需求明显的患者，则应尽早转入积极干预路径^[13,24]^。基于上述评估要点，肺癌术后声音嘶哑的临床处理可概括为由症状识别、病因判断到分层干预与动态随访的连续路径（附图1， http://www.lungca.org/files/2026s111/2026s111-s1.pdf）。

## 4 治疗与综合管理

### 4.1 治疗决策应基于症状严重程度、误吸风险及恢复预期

肺癌术后声音嘶哑的处理目标在于改善发声功能并降低误吸相关结局风险，同时兼顾有效咳嗽、气道保护以及交流能力与生活质量^[5,8,13]^；因此，治疗决策需结合声带麻痹侧别与声门闭合程度、吞咽功能及呼吸道并发症风险，并综合考虑神经恢复可能性^[13,23]^。症状轻微、无明显误吸且具恢复前景者可先行密切随访与阶段性保守处理；若出现明显发声障碍、反复饮水呛咳或肺部感染倾向，则应尽早进入积极干预以避免功能持续受损。同时，还需将肿瘤治疗时间轴纳入声音恢复的综合评估。肺癌术后辅助化疗、免疫治疗、靶向治疗及放疗可能通过影响黏膜炎症、水肿、诱发或加重免疫相关神经炎或周围神经病变，以及促进局部纤维化等机制，间接改变声带麻痹恢复进程与临床表现；但目前关于其对声带麻痹恢复的直接高质量证据仍有限，因此不宜将恢复差异简单归因于手术损伤或药物不良反应。临床上，若辅助治疗期间出现新发或加重的声音改变、吞咽困难或误吸风险升高、呼吸困难或神经肌无力样表现，应结合喉镜，必要时行神经系统评估并整合影像学及用药史进行综合判断与个体化干预。

### 4.2 嗓音训练是基础干预措施，宜尽早启动

嗓音训练是单侧声带麻痹综合治疗的重要组成部分，尤其适用于术后早期、中轻度或作为介入治疗前后辅助康复的患者^[13,25]^。规范的言语治疗可通过改善呼吸-发声协调、减少异常代偿发声及提高声门闭合效率，在一定程度上缓解发声费力、音量不足及嗓音疲劳等问题^[13,25]^。对于肺癌术后患者而言，早期嗓音康复除有助于改善发声功能外，理论上还可能通过改善声门闭合和呼吸-发声协调而间接改善有效咳嗽与气道保护，因此不宜将其仅视为“声音美观”的辅助治疗。

### 4.3 注射喉成形术可作为早期改善声门闭合的重要手段

对于存在明显声门闭合不全、声音障碍显著或误吸风险较高的患者，注射喉成形术是一种微创、起效较快的干预方式^[4,26,27]^。该方法通过增加患侧声带体积、改善声门闭合，可在较短时间内改善发声功能，并有助于减轻吞咽相关症状及改善生活质量^[4,27]^。动物模型研究^[28]^亦提示早期注射可改善受损声带的生物力学特性并延缓肌萎缩。目前基于回顾性队列研究及病例系列分析^[4,26,27,29]^的结果，提示早期注射喉成形或其他以改善声门闭合为目的的内移化干预，可改善发声和吞咽相关功能，并可能与短期及长期肺炎发生率下降有关。然而，该治疗不应被理解为适用于所有早期声带麻痹患者。术后早期声带运动障碍中，部分可能与神经传导阻滞、牵拉性神经损伤、局部水肿或炎症反应有关，具有一定自发恢复可能^[6,7]^。因此，对于症状轻微、无明显声门闭合不全、无误吸风险、无排痰困难且评估提示仍存在恢复可能的患者，可优先采取观察、嗓音治疗与吞咽康复，并在随访中动态评估声带运动恢复趋势^[21,22]^。早期注射喉成形术更适用于：严重声音嘶哑显著影响交流或生活质量者；喉镜提示明显声门闭合不全者；合并饮水呛咳、误吸风险、排痰无力或反复肺部感染倾向者；以及职业用声需求较高、短期内需恢复发声功能的患者^[21,22,30]^。

### 4.4 永久性手术应在恢复预期明确后审慎实施

对于经随访观察后仍无明显恢复、症状持续存在且功能受损显著的患者，可考虑甲状软骨成形术、杓状软骨内收术或喉神经再支配等永久性手术治疗^[13,24]^。此类手术在改善声门闭合、提高发声稳定性及减少误吸方面通常具有较明确的功能获益，但通常应建立在神经功能恢复可能性较低、病情相对稳定及患者获益预期明确的基础上^[13,31]^。对于肺癌术后患者，是否实施永久性手术还应结合肿瘤分期、后续抗肿瘤治疗安排、全身耐受状态及预后综合判断，以避免治疗目标与整体肿瘤管理策略脱节。

### 4.5 多学科协作有助于提升整体管理效果

肺癌术后声音嘶哑的管理不应局限于单一专科。胸外科、耳鼻喉科、言语治疗师、康复医学科及护理团队之间的协作，有助于实现从病因识别、功能评估到干预实施和随访管理的闭环^[4,13]^。对于合并吞咽障碍、反复误吸、排痰无力或肺部感染的患者，多学科模式尤为重要。通过建立标准化转诊流程与分层干预路径，可实现从病因识别、功能评估到干预与随访的管理闭环，减少漏诊与处置不一致。

## 5 预防策略

### 5.1 术前风险识别与手术规划优化

术前应结合影像学资料识别高危解剖关系及潜在变异。对于预计需行左侧纵隔系统性淋巴结清扫、主动脉弓下操作范围较大或局部粘连明显的患者，应重点评估喉返神经相关解剖风险^[1-3,15]^。4L转移并非罕见，其4L清扫在左侧肺癌中具有明确的临床意义^[16-18]^，可能带来长期生存获益。Wang等^[16]^对左侧肺癌手术患者进行回顾性比较，发现4L区转移率为20.9%，且行4L清扫者的长期生存优于未行者；5年无病生存（disease-free survival, DFS）率分别为54.8%和42.7%，5年总体生存（overall survival, OS）率分别为58.9%和47.2%；在多因素及倾向评分加权分析后，4L清扫仍表现出独立的有利生存效应。此外，Deng等^[17]^的meta分析纳入倾向评分匹配的回顾性队列，进一步支持4L清扫与生存获益有关：与未行4L清扫相比，4L清扫可提高5年OS率（分别为67.7%和54.6%）与5年DFS率（分别为53.3%和44.8%），并在校正后仍显示OS及DFS获益。对于分期价值，Hanaoka等^[18]^在左上叶肺癌队列中观察到4L转移可在部分经纵隔镜评估为阴性的患者中出现，提示系统的4L区清扫有助于识别隐匿性病灶，从而提高分期与治疗决策的准确性。术前风险识别的关键并非简单缩小清扫范围，而是结合影像与局部解剖特征制定更精细的神经保护策略。

### 5.2 精细化操作与能量器械控制是核心

术中神经保护的关键在于清晰辨认解剖层次、减少不必要的神经暴露与牵拉，并控制神经邻近区域的热损伤^[2,10,11]^。在4L清扫时鉴于4L区域与左喉返神经毗邻程度高，应避免粗暴牵拉、长时间近距离使用高热能量器械及过度骨骼化分离^[10,11]^。与此同时，在右侧气管旁淋巴结清扫过程中，尤其当存在淋巴结肿大融合、炎症粘连、新辅助治疗后纤维化或解剖层次不清等情况时，亦应采取精细锐性分离、减少过度牵拉及限制能量器械的使用等^[3,16,32]^。此外，新辅助治疗后纤维化、炎症粘连及局部瘢痕反应也可能进一步改变神经周围组织微环境，使神经更易受牵拉与间接应力影响^[6,16,32]^。

关于4L清扫时是否应常规暴露左喉返神经，目前仍存在一定争议：基于现有证据，较为稳妥的策略并非在所有病例中机械性全程裸化左喉返神经，而是在明确4L清扫解剖边界的基础上，主动判断神经可能走行，并在关键高危区域进行必要的显露与保护；对于低风险、淋巴结未明显肿大且解剖层次清晰的病例，可沿淋巴结包膜及正确解剖层面进行精细锐性分离，尽量避免对神经主干的过度游离；对于高风险病例，如4L融合粘连、靠近气管食管沟、主动脉弓下方结构显示不清、既往治疗导致组织纤维化或术中需要较大范围牵拉时，应主动识别左迷走神经及左喉返神经走行^[2,14,20,33]^。目前相关研究^[10,11]^还提示，采用非能量器械或减少高热源暴露有助于降低喉返神经损伤风险。

### 5.3 持续术中神经监测（continuous intraoperative neuromonitoring, C-IONM）在高危病例中具有潜在价值

对于预计解剖关系复杂、需行广泛上纵隔或主动脉弓下清扫的高危病例，持续术中神经监测C-IONM，可作为辅助性手段，用于帮助识别神经走行、评估神经功能状态，并增强术者对高风险操作步骤的实时警觉^[2,34-36]^；然而，甲状腺外科领域的系统综述与meta分析提示，其在降低喉返神经损伤发生率方面的增量获益尚缺乏一致结论^[37]^。例如，Seong等^[2]^在左侧电视辅助胸腔镜手术（video-assisted thoracoscopic surgery, VATS）肺叶切除联合4L清扫的研究中观察到，未行C-IONM组术后声带麻痹发生率为22.7%，C-IONM组为20.0%；但永久性声带麻痹发生率分别为 22.7%和5.0%，提示C-IONM可能有助于降低永久性喉返神经损伤风险。Chai等^[20]^亦认为C-IONM可实时识别神经功能变化，为及时调整操作策略提供依据。尽管，目前有关C-IONM在肺癌手术中应用价值的直接证据虽已有报道，但总体仍相对有限^[2,36]^，部分依据仍需借鉴甲状腺及食管外科研究结果^[34,35]^；因此，其在胸外科中的作用目前更宜定位为高危病例中的选择性辅助工具，而非适用于所有患者的常规策略。

## 6 总结与展望

肺癌术后声音嘶哑是胸外科围术期较常见而又容易被低估的并发症之一，其核心机制多与喉返神经损伤有关，尤以左侧肺癌手术及4L清扫后更值得关注。然而，术后声音改变并不完全等同于喉返神经麻痹，临床上仍需注意与气管插管相关喉部损伤、杓状软骨脱位及其他可逆性病变相鉴别。由于该并发症不仅影响发声质量，还可能进一步削弱有效咳嗽、吞咽协调及气道保护功能，因此其临床意义不应局限于“声音问题”本身。

肺癌术后声音嘶哑的管理应贯穿术前、术中和术后全过程。术前应结合影像学资料识别高危解剖关系及潜在变异；术中应重视喉返神经走行判断、解剖层次辨识与功能保护，尽量减少过度牵拉、压迫及热损伤；术后则应尽早识别高危症状，并联合喉镜检查、嗓音功能评估、吞咽功能评估及必要的喉肌电图，完成病因判断与功能分层。在此基础上，根据症状严重程度、误吸风险、恢复预期及患者用声需求，采取观察随访、嗓音治疗、吞咽康复、注射喉成形或进一步外科干预等个体化策略，有助于改善患者功能结局和生活质量。

总体而言，肺癌术后声音嘶哑证据主要来自回顾性分析与单中心经验，缺乏胸外科人群统一的结局定义与分层标准。未来应通过可推广的前瞻性路径提升证据水平：（1）多中心建立术后声音嘶哑及喉返神经损伤登记系统，对纵隔清扫以及4L清扫范围、术中神经监测方式与参数、术后喉镜分级、吞咽与误吸结局，以及辅助化疗、免疫治疗、靶向治疗与放疗时间轴进行标准化采集；（2）针对单侧声带麻痹开展前瞻性研究，必要时实施对照试验（如注射喉成形术与嗓音训练对比），以声带活动度、喉镜分级、VHI、误吸或肺部感染以及生活质量为主要结局，界定不同恢复阶段的最佳干预窗口；（3）比较不同手术平台或神经保护策略（如机器人手术与VATS或开胸）对神经损伤及功能恢复的影响，并在标准化框架下评估潜在优势能否转化为可重复的临床获益。
